# Cut-off points of the Portuguese version of the Montreal Cognitive Assessment for cognitive evaluation in Parkinson’s disease

**DOI:** 10.1590/1980-57642018dn13-020010

**Published:** 2019

**Authors:** Kelson James Almeida, Larissa Clementino Leite de Sá Carvalho, Tomásia Henrique Oliveira de Holanda Monteiro, Paulo Cesar de Jesus Gonçalves, Raimundo Nonato Campos-Sousa

**Affiliations:** 1 Universidade de São Paulo Faculdade de Medicina Ringgold standard institution São Paulo, SP, Brazil.; 2 FACID Curso de Medicina Ringgold standard institution, Teresina, PI Brazil.; 3 Universidade Federal do Piaui Ringgold standard institution Teresina, Piauí Brazil.

**Keywords:** Parkinson’s disease, MOCA-BR, dementia in Parkinson’s disease (PD-D), mild cognitive impairment (PD-MCI), cognitive assessment, doença de Parkinson, MOCA-BR, demência na doença de Parkinson (DP-D), comprometimento cognitivo leve (DP-CCL), avaliação cognitiva

## Abstract

The Movement Disorder Society has published some recommendations for dementia diagnosis in Parkinson disease (PD), proposing the Montreal Cognitive Assessment (MOCA) as a cognitive screening tool in these patients. However, few studies have been conducted assessing the Portuguese version of this test in Brazil (MOCA-BR).

**Objective:**

the aim of the present study was to define the cut-off points of the MOCA-BR scale for diagnosing Mild Cognitive Impairment (PD-MCI) and Dementia (PD-D) in patients with PD.

**Methods:**

this was a cross-sectional, analytic field study based on a quantitative approach. Patients were selected after a consecutive assessment by a neurologist, after an extensive cognitive evaluation, and were classified as having normal cognition (PD-N), PD-MCI or PD-D. The MOCA-BR was then applied and 89 patients selected.

**Results:**

on the cognitive assessment, 30.3% were PD-N, 41.6% PD-MCI and 28.1% PD-D. The cut-off score on the MOCA-Br to distinguish PD-N from PD-D was 22.50 (95% CI 0.748-0.943) for sensitivity of 85.5% and specificity of 71.1%. The cut-off for distinguishing PD-D from MCI was 17.50 (95% CI 0.758-0.951) for sensitivity of 81.6% and specificity of 76%.

## RESUMO.

A *Movement Disorder Society* publicou algumas recomendações para o diagnóstico de demência na doença de Parkinson (DP), propondo o *Montreal Cognitive Assessment* (MOCA) como ferramenta de triagem cognitiva nesses pacientes. Entretanto, poucos estudos foram aplicados à versão em português (MOCA-BR).

**Objetivo:**

o presente estudo tem o objetivo de definir os valores de corte na escala de MOCA-BR para diagnosticar o Comprometimento Cognitivo Leve (DP-CCL) e Demência (DP-D) em pacientes com DP.

**Métodos:**

trata-se de um estudo transversal, analítico, com uma abordagem quantitativa. Os pacientes foram selecionados depois de avaliações consecutivas por um neurologista, após avaliação cognitiva extensa, e foram classificados como cognição normal (DP-N), DP-CCL e DP-D e então o MOCA-BR foi aplicado, sendo selecionados 89 pacientes.

**Resultados:**

na avaliação cognitiva, foram encontrados 30.3% de DP-N, 41.6% de DP-CCL e 28.1% DP-D. O valor de corte no MOCA-BR para distinguir entre DP-N de DP-D foi 22.5 (IC 95%; 0.748-0.943), sensibilidade de 85.5% e especificidade de 71.1%. Para distinguir DP-P de CCL, o ponto de corte foi de 17.5 (IC 95%; 0.758-0.951), sensibilidade de 81.6% e especificidade de 76%.

Among non-motor manifestations, cognitive impairment is recognized as a common component of Parkinson disease (PD) and includes dementia (PD-D) and mild cognitive impairment (PD-MCI). Cognitive impairment is associated with decreased quality of life, increased functional disability, caregiver distress and institutionalization.^1^^-^ 6

The Movement Disorder Society Task Force recommends the Montreal Cognitive Assessment (MOCA) as a minimal standard or cognitive screening instrument in PD clinical trials.^3^^,^ 7 The capacity to detect executive dysfunction in PD is the main advantage of MOCA over the Mini-Mental State Examination (MMSE), a widely used test in cognitive screening, but which fails to effectively assess executive function, the domain initially affected in PD.^8^^-^ 12

However, there are few studies using the Portuguese version of MOCA (MOCA-BR) to determine cut-off scores of this test for PD-MCI and PD-D diagnosis.^13^^-^ 16

The aim of this study was to define the cut-off points on the MOCA-BR scale for diagnosing Mild Cognitive Impairment and Dementia in patients with PD in comparison to an extensive neuropsychological evaluation.

## METHODS

This is a descriptive, cross-sectional, observational, analytical and quantitative field study.

### Ethical procedures

The study was submitted and registered on Plataforma Brasil and was only initiated after review and approval by the Facid-Devry Research Ethics Committee (FACID-REC) (CAAE No.: 64825416.1.0000.5211). The subjects who agreed to participate in this study signed the Informed Consent Form (ICF) after having received study information and clarification.

### Setting and study subjects

The study was carried out at a neurological health facility which is a referral center for movement disorders, located in the city of Teresina, Piauí state. The random sample was statistically calculated using a sample calculator to obtain a significance level of 0.05 and a confidence level of 0.95.

Patients were recruited consecutively during 2016, until the calculated sample size had been reached. All patients were then assessed by two neurologists who are experts in Movement Disorders, and by a neurologist who is an expert in cognition. Patients diagnosed with Parkinson’s disease according to the London Brain Bank and who had a brain magnetic resonance imaging scan with no changes were selected.^17^

The inclusion and exclusion criteria of this study were:

- Inclusion criteria: diagnosis of idiopathic Parkinson’s disease according to the London Brain Bank criteria associated with age over 45 years, no changes on brain magnetic resonance imaging scan and no red flags for atypical Parkinson’s disease.
- Exclusion criteria: comorbidities associated with Parkinson’s disease that can interfere with the cognitive assessment; positive screening for major depression according to Beck’s Depression Inventory (BDI); patients who have undergone neurosurgery; early onset Parkinson’s disease and other types of Parkinsonism.

### Data collection

Neuropsychological tests were previously structured by a neurologist specialized in cognitive neurology. This study used a neuropsychological battery based on level II criteria proposed by the Movement Disorder Society Task Force for PD cognition assessment. Following these recommendations, a comprehensive cognitive assessment was performed involving at least two tests for each of the five cognitive domains.^15^

These tests were applied during a 40-minute session, depending on the level of difficulty of each patient. First, a study-adapted questionnaire was applied with clinical and epidemiological variables. Tests were then used to assess patients’ cognitive domains and global cognition:

The Mini-Mental State Examination and MOCA-BR were used to assess global cognition. The following tests were used to assess each cognitive domain: Memory (Brief Cognitive Battery, Semantic fluency), Attention and Executive Function (Trail Making Test (TMT-B), Direct Digit Span, Reverse Digit Span and Clock Test), Language (Oral fluency and Semantic fluency), and Visuospatial skills (Clock Drawing Test and Pentagon Test). For the analysis of the performance on the TMT-B and for Visuospatial skills, the results were categorized as a wrong or correct complete task.

Cognitive tests were reviewed by the same cognition expert and patients subsequently classified into 3 groups: Parkinson’s disease with normal cognition, Parkinson’s disease with mild cognitive impairment, and Parkinson’s disease with dementia. The PD-MCI and PD-D classification was based on the level II criteria of the Movement Disorder Society.^15^

Subsequently, cognitive assessment results were compared to those obtained on the MOCA-BR screening test (level I criteria) to calculate cut-off scores for this latter assessment.^15^ MOCA scores were then used to calculate the cut-off points for PD-MCI and PD-D after an extensive neuropsychological evaluation.

All subjects were screened to rule out major depression using Beck’s Depression Inventory (BDI). Functional disability was assessed by the Pfeffer Functional Assessment Scale (PFEFFER) and the Informant Questionnaire on Cognitive Decline in the Elderly (IQCODE), adapted for use in Brazil.

### Organization and data analysis

Classified data were organized in four files with the same variables: PD-N, PD-MCI, PD-D, and all patients together. Data collected from questionnaires were organized using the Statistical Package for Social Sciences software (IBM SPSS *Statistics*), version 21.0.

Subsequently, when all the information had been interpreted, data were tabulated with absolute frequency, means, standard deviation, and minimum and maximum intervals. Student’s *t-*test, the Chi-squared test, Kruskal-Wallis test, and ANOVA were used. A 95% confidence interval was defined and a p-value ≤ 5% adopted to exclude the null hypothesis.

Subsequently, cognitive assessment results were compared against those obtained on the MOCA-BR screening test (level I criteria) to calculate cut-off scores for the scale which distinguished patients in the groups in question using ROC Curve analysis.^15^

## RESULTS

Of the 115 subjects recruited from the calculated sample, 26 were excluded for the following reasons: positive screening for major depression (n=13), early onset PD(n=8), others types of parkinsonism (n=5). Thus, the final sample comprised 89 patients. Out of these, 46.07% were female and 53.93% male. The mean age was 63.52 years, ranging from 47 to 85 years.

### Sample characteristics

The clinical and demographic characteristics of subjects in this study are given in Table 1. Patients were divided into three groups according to their cognitive status, based on the Level II criteria proposed by the Movement Disorder Society Task Force.^7^

**Table 1:** Clinical and demographic characteristics of Parkinson’s disease patients.

	PD-N (n = 26)	PD-MCI (n = 38 )	PD-D (n = 25 )	P-value
Age	57.15 ± 7.81	66.53 ± 8.64	65.48 ± 8.67	<0.001^a^
Gender (M/F)	19/7	18/20	11/14	0.021^b^
Education (years)	11.15 ± 4.7	8.74 ± 5.54	5.63 ± 4.68	<0.001^a^
Disease duration (months)	59.88 ± 46.69	66.97 ± 59.33	78.83 ± 68.76	0.791^a^
BDI	5.38 ± 5.47	9.03 ± 4.95	11.33 ± 4.84	<0.001^a^
MMSE	27.42 ± 2.26	24.13 ± 3.21	20.56 ± 4.75	<0.001^a^
MOCA-BR	25.42 ± 3.13	20.76 ± 3.67	15.28 ± 3.6	<0.001^a^

PD-N: Parkinson’s disease with normal cognition; PD-MCI: Parkinson’s disease with mild cognitive impairment; PD-D: Parkinson’s disease with dementia; BDI: Beck’s Depression Inventory; MMSE: Mini-Mental State Examination; MOCA-BR: Montreal Cognitive Assessment adapted to Brazilian Portuguese;

a ANOVA;

b Likelihood Ratio (Chi-square).

For mean age and disease duration, patients with PD-MCI and PD-D had a higher mean age than that of PD-N patients, where this difference was statistically significant. The mean age of the PD-D patients was 65.48 ± 8.67 years. Mean age was higher in the group with worse cognitive performance.

PD-D patients had lower mean educational level than PD-MCI and PD-N patients, where this was statistically significant. Only 13 patients in this study (14.6%) had less than 4 years of education. Regarding these patients, 1 patient was classified as PD-N, 3 as PD-MCI and 9 as PD-D. Even in the PD-D group, the mean years of education was ≥4 years (5.63 ± 4.68 years). PD patients with normal cognition had a higher educational level.

Regarding PD patients’ performance on the MMSE, PD-MCI patients scored lower than the cut-offs scores proposed for the test, even when assessed on a case-by-case basis compared to corresponding educational level.

### Cognitive performance of Parkinson’s disease patients

PD-D and PD-MCI patients performed worse on all tests assessing executive function compared to PD-N patients, a difference that reached statistical significance. Additionally, the TMT B test was the instrument on which the PD-D patients had worse impairment, compared with the other tests used to evaluate executive function. Only one patient classified with PD-D was able to perform the test correctly.

For visuospatial skills, worse performance was observed on this domain, with a statistically significant difference for PD-MCI patients and, more markedly, for PD-D patients. On the Pentagon Test, only one patient classified as PD-N was unable to perform the test correctly.

**Table 2:** Cognitive performance of Parkinson’s disease patients.

		Median			P-value
		PD-N (n = 26)	PD-MCI (n = 38)	PD-D (n = 25)	
Attention/Executive Function	Digit Span (Direct)	5.62 ± 0.98	5.05 ± 0.98	4.68 ± 1.24	0.011^a^
	Digit Span (Reverse)	3.62 ± 1.09	2.84 ± 0.75	2.12 ± 0.97	<0.001^b^
	TMT B	20/3	11/26	1/23	<0.001^c^
Memory	Brief Cognitive Battery (delayed recall)	8.46 ± 1.3	7.13 ± 1.96	6.20 ± 2.02	0.004^c^
Language	Semantic Fluency (Animals)	14.77 ± 3.93	12.13 ±3.84	9.80 ± 4.28	0.005^b^
	Phonemic Fluency (Letter P)	10.92 ± 5.21	8.29 ± 4.89	4.24 ± 3.64	0.002^b^
Visuospatial Skills	Clock Drawing Test	7.43 ± 2.35	5.03 ± 2.87	3.58 ± 2.33	0.001^c^
	Pentagon Test	23/1	22/14	4/18	<0.001^c^
Functional Activity	PFEFFER	1.20 ± 1.17	2.03 ± 2.27	8.33 ± 6.30	<0.001^b^
	IQCODE	2.00 ± 1.4	3.24 ± 0.22	3.64 ± 0.40	<0.001^b^

PD-N; Parkinson’s disease with normal cognition; PD-MCI; Parkinson’s disease with mild cognitive impairment; PD-D; Parkinson’s disease with dementia; TMT B; Trail Making Test B; PFEFFER; Pfeffer Functional Assessment Scale; IQCODE; Informant Questionnaire on Cognitive Decline in the Elderly;

a ANOVA;

b Kruskal-Wallis;

c Likelihood Ratio (Chi-square);

TMT and visuospatial (adequate/inadequate).

**Figure 1:**
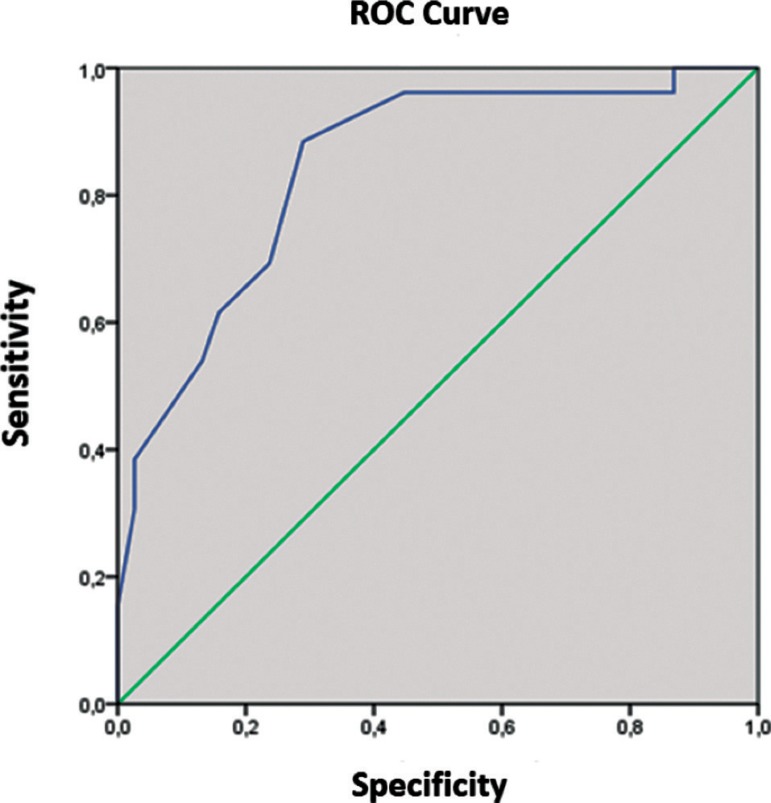
ROC curve determining cut-off score to distinguish Parkinson’s disease patients with normal cognition from patients with mild cognitive impairment using the MOCA-BR.

**Figure 2:**
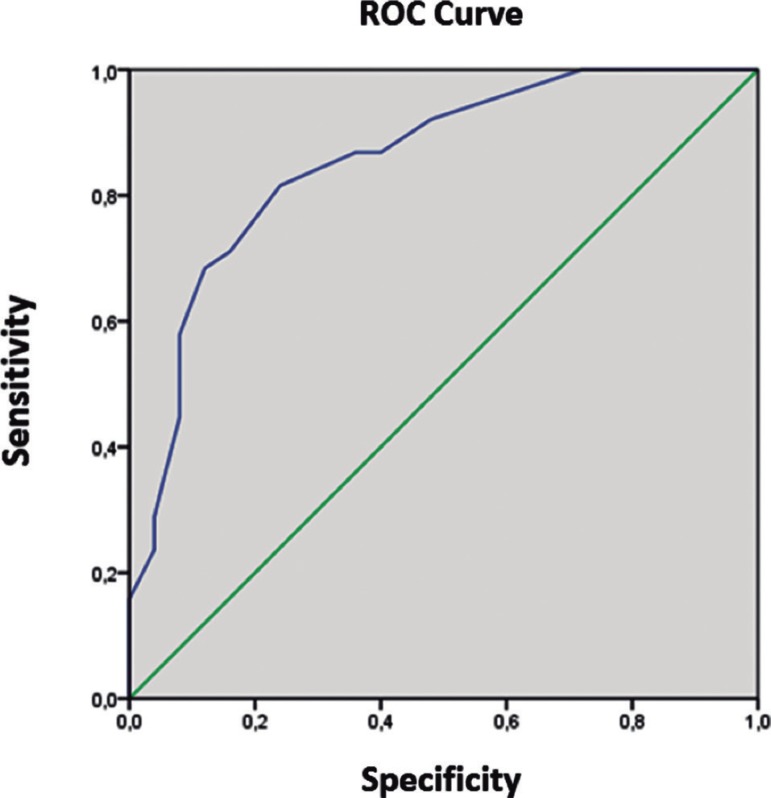
ROC curve determining cut-off score to distinguish Parkinson’s disease patients with mild cognitive impairment from patients with dementia using the MOCA-BR.

Memory performance was worse in PD-D patients compared to patients without dementia, where this reached statistical significance.

For the language cognitive domain, the study subjects performed well on the semantic fluency test , but worse on the phonemic fluency test. The mean score of patients in all groups was below the cut-off value. This pattern in phonological verbal fluency represents dysexecutive aspects, rather than language aspects.

### Cut-off scores on MOCA-BR scale

In this study, the cut-off score obtained on the MOCA-BR to distinguish PD-N from PD-MCI patients was 22.50, with 84.5% accuracy (95% CI 0.748-0.943, p < 0.001), 85.5% sensitivity and 71.1% specificity. The cut-off score to distinguish PD-MCI from PD-D patients was 17.50, with 85.5% accuracy (95% CI 0.758-0.951, p < 0.001), 81.6% sensitivity and 76% specificity.

## DISCUSSION

The MOCA was originally designed to screen mild cognitive impairment (MCI) in the general population.^4^^,^ 8 ^,^ 11 ^,^ 12 This test has been translated and adapted for clinical use in Brazil. Using a cut-off score of 25/26, this test has 80-100% sensitivity and 50-76% specificity for detecting MCI.^4^^,^ 13 ^,^ 14 The ability to detect executive dysfunction in PD is the main advantage of the MOCA over the Mini-Mental State Examination (MMSE). This has been frequently attributed to the limited executive function assessment of the MMSE, a domain frequently affected in PD.^13^^,^ 14 ^,^ 16 ^,^ 18

Several studies have shown that the MOCA has high sensitivity when compared to the several cognitive screening tests available for MCI diagnosis in a number of populations, not only in PD.^9^^,^ 11 ^,^ 19 ^-^ 21 A systematic review assessing the diagnostic performance of all cognitive tests for detecting of dementia due to several etiologies, including PD, concluded that the MOCA performed better than the other tests in detecting MCI.^20^ Another study comparing the usefulness and diagnostic accuracy of the MOCA and the MMSE in diagnosing Alzheimer’s disease and MCI, concluded that the MOCA proved more sensitive and accurate for diagnosing MCI.^10^

The MOCA was translated and adapted for clinical use in Brazil (MOCA-BR). Two important Brazilian studies have used the MOCA-BR. The first study was conducted to validate the MOCA-BR for diagnosing mild cognitive impairment in the elderly from the general population. The cut-off score obtained for the MOCA-BR to distinguish normal patients from MCI patients was 25 points, with 81% and 77% sensitivity and specificity, respectively.^22^ In the second study, the MOCA-BR was used to diagnose MCI and dementia in 79 patients with PD. In this study, the cut-off score obtained for the MOCA-BR to distinguish normal patients from PD-MCI patients was 26, with 84% sensitivity and 27% specificity. To distinguish PD-MCI patients from PD-D patients, the cut-off score was 21, with 94% sensitivity and 68% specificity.^16^

When comparing the results of Sobreira et al.to those of the present study, the mean educational level of PD-D patients in both studies was quite similar, 5.50 (2-18) and 5.63 (0-16), respectively.^16^ However, there was considerable divergence when comparing the cut-off scores obtained. This might be due to the difference in the number of patients classified as PD-D in the two studies. In the study by Sobreira et al., the number of patients with PD-D (17) was much lower than the number of patients with PD-N (30) and PD-MCI (32), which may have influenced the cut-off score obtained. By contrast, the present study had a more homogenous number of patients across the groups: PD-N (26), PD-MCI (38), and PD-D (25).^16^ Additionally, this study was carried out in a different region of Brazil from the other study that assessed MOCA-BR in this specific population.^18^

In conclusion, the cut-off scores on the MOCA-BR scale for diagnosing PD-MCI and PD-D were 22.50 and 17.50, respectively. The prevalence of PD-MCI was 41.6% and of PD-D was 28.1%.

The contribution of this study is that the cut-off scores obtained on the MOCA-BR, both to distinguish PD-N from PD-MCI and PD-MCI from PD-D, have high sensitivity and specificity. Therefore, these cut-off scores can be used to screen mild cognitive impairment and dementia in patients with PD.

A limitation of this study was that 14% of the patients had an educational level of less than 4 years. However, there are other studies which assessed the MOCA in patients with Parkinson’s disease that had a lower educational level.^19^ Also, these patients were not assessed for clinical staging. However, the subjects in this study had the disease for more than 60 months, and this enabled accurate diagnosis of parkinsonism etiology. The differences in educational level among participants in the different groups may have influenced some of the cognitive outcomes of this study. Moreover, the number of patients in each group was not the same, but relatively homogeneous compared to other studies published on this subject.

Therefore, considering the high prevalence of cognitive disorders in PD, further studies using the MOCA-BR and its respective cut-off scores are necessary in different regions of Brazil.

## References

[1] Aarsland D, Zaccai J, Brayne C (2005). A systematic review of prevalence studies of dementia in
Parkinson's disease. Mov Disord.

[2] Aarsland D, Bronnick K, Williams-Gray C, Weintraub D, Marder K, Kulisevsky J (2010). Mild cognitive impairment in Parkinson disease: a multicenter
pooled analysis. Neurology.

[3] Brown DS, Bernstein IH, McClintock SM, Munro CC, Dewey RBJ, Husain M (2016). Use of the Montreal Cognitive Assessment and Alzheimer's
Disease-8 as Cognitive Screening Measures in Parkinson
Disease. Int J Geriatr Psychiatry.

[4] Burdick DJ, Cholerton B, Watson GS, Siderowf A, Trojanowski JQ, Weintraub D (2014). People with Parkinson's disease and normal MMSE score have a
broad range of cognitive performance. Mov Disord.

[5] Leung IH, Walton CC, Hallock H, Lewis SJ, Valenzuela M, Lampit A (2015). Cognitive training in Parkinson disease: a systematic review and
meta-analysis. Neurology.

[6] Marras C, Armstrong MJ, Meaney CA, Fox S, Rothberg B, Tang-Wai DF (2013). Measuring Mild Cognitive Impairment in Patients With Parkinson's
Disease. Mov Disord.

[7] Hu MT, Szewczyk-Królikowski K, Tomlinson P, Nithi K, Rolinski M, Murray C (2014). Predictors of cognitive impairment in an early stage Parkinson's
disease cohort. Mov Disord.

[8] Nasreddine ZS, Philips NA, Bédirian V, Charbonneau S, Whitehead V, Collin I (2005). The Montreal Cognitive Assessment, MoCA: a brief screening tool
for mild cognitive impairment. J Am Geriatr Soc.

[9] Rambe AS, Fitri FI (2017). Correlation between the Montreal Cognitive Assessment-Indonesian
Version (Moca-INA) and the Mini-Mental State Examination (MMSE) in
Elderly. Open Access Maced J Med Sci.

[10] Roalf DR, Moberg PJ, Xie SX, Wolk DA, Moelter ST, Arnold SE (2013). Comparative accuracies of two common screening instruments for
classification of Alzheimer's disease, mild cognitive impairment, and
healthy aging. Alzheimers Dement.

[11] Roalf DR, Moore TM, Wolk DA, Arnold SE, Mechanic-Hamilton D, Rick J (2016). Defining and validating a short form Montreal Cognitive
Assessment (s-MoCA) for use in neurodegenerative disease. J Neurol Neurosurg Psychiatry.

[12] Trzepacz PT, Hochsteller H, Wang S, Walker B, Saykin AJ (2015). Relationship between the Montreal Cognitive Assessment and
Mini-mental State Examination for assessment of mild cognitive impairment in
older adults. BMC Geriatr.

[13] Chou KL, Amick MM, Brandt J, Camicioli R, Frei K, Gitelman D (2010). A recommended scale for cognitive screening in clinical trials of
Parkinson's disease. Mov Disord.

[14] Hoops S, Nazem S, Siderowf AD, Duda JE, Xie SX, Stern MB (2009). Validity of the MoCA and MMSE in the detection of MCI and
dementia in Parkinson disease. Neurology.

[15] Litvan I, Goldaman JG, Troster AI, Schmand BA, Weintraub D, Petersen RC (2012). Diagnostic criteria for mild cognitive impairment in Parkinson's
disease: Movement Disorder Society Task Force Guidelines. Mov Disord.

[16] Sobreira E, Pena-Pereira MA, Eckeli AL, Sobreira-Neto MA, Chagas MH, Foss MP (2015). Screening of cognitive impairment in patients with Parkinson's
disease: diagnostic validity of the Brazilian versions of the Montreal
Cognitive Assessment and the Addenbrooke's Cognitive
Examination-Revised. Arq Neuropsiquiatr.

[17] Jankovic J (2008). Parkinson's disease: clinical features and
diagnosis. J Neurol Neurosurg Psychiatry.

[18] Langa KM, Levine DA (2014). The Diagnosis and Management of Mild Cognitive Impairment: A
Clinical Review. JAMA.

[19] Kim JI, Sunwoo MK, Sohn YH, Lee PH, Hong JY (2016). The MMSE and MoCA for Screening Cognitive Impairment in Less
Educated Patients with Parkinson's Disease. J Mov Disord.

[20] Tsoi KK, Chan JY, Hirai HW, Wong SY, Kwok TC (2015). Cognitive Tests to Detect Dementia: A Systematic Review and
Meta-analysis. JAMA Intern Med.

[21] Velayudhan L, Ryu SH, Raczek M, Philpot M, Lindesay J, Critchfield M, Livingston G (2014). Review of brief cognitive tests for patients with suspected
dementia. Int Psychogeriatr.

[22] Memoria CM, Yassuda MS, Nakano EY, Forlenza OV (2013). Brief screening for mild cognitive impairment: validation of the
Brazilian version of the Montreal cognitive assessment. Int J Geriatr Psychiatry.

